# Myocardial Protection from Ischemia-Reperfusion Damage by the Antioxidant Effect of *Hibiscus sabdariffa Linnaeus* on Metabolic Syndrome Rats

**DOI:** 10.1155/2019/1724194

**Published:** 2019-03-26

**Authors:** Israel Pérez-Torres, Juan Carlos Torres-Narváez, Verónica Guarner-Lans, Eulises Díaz-Díaz, Mario Perezpeña-Diazconti, Andrea Romero Palacios, Linaloe Manzano-Pech

**Affiliations:** ^1^Department of Pathology, Juan Badiano 1, Sección XVI, Tlalpan, Instituto Nacional de Cardiología “Ignacio Chávez”, México City 14080, Mexico; ^2^Department of Pharmacology, Juan Badiano 1, Sección XVI, Tlalpan, Instituto Nacional de Cardiología “Ignacio Chávez”, México City 14080, Mexico; ^3^Department of Physiology, Juan Badiano 1, Sección XVI, Tlalpan, Instituto Nacional de Cardiología “Ignacio Chávez”, México City 14080, Mexico; ^4^Department of Reproductive Biology, Vasco de Quiroga 15, Sección XVI, Tlalpan, Instituto Nacional de Ciencias Médicas y Nutrición “Salvador Zubirán”, México City 14000, Mexico

## Abstract

Cardiovascular diseases (CVD) constitute one of the most prevalent health problems worldwide, being strongly associated with metabolic syndrome (MS). Oxidative stress (OS) is present in both CVD and MS. Infusions of *Hibiscus sabdariffa Linnaeus* (HSL) have antioxidant properties and could therefore decrease the presence of OS in these diseases. The aim of this study was to evaluate myocardial protection during ischemia/reperfusion due to the antioxidant effect of HSL infusion (3%) on a MS rat model induced by the administration of 30% sucrose in drinking water. We determined in control, MS, and MS + HSL rat hearts (*n* = 6 per group) cardiac mechanical performance (CMP), coronary vascular resistance (CVR), and activities of manganese and copper/zinc superoxide dismutases (Mn and Cu/Zn-SOD), peroxidases, glutathione peroxidase (GPx), catalase (CAT), glutathione s-transferase (GST), glutathione reductase (GR), and glutathione (GSH). We also determined lipoperoxidation (LPO), total antioxidant capacity (TAC), and the nitrate/nitrite ratio (NO_3_^−^/NO_2_^−^). The treatment with the HSL infusion restored the CMP (*p* = 0.01) and CVR (*p* = 0.04) and increased the Mn- (*p* = 0.02), Cu/Zn-SOD (*p* = 0.05), peroxidases (*p* = 0.04), GST (*p* = 0.02) activity, GHS (*p* = 0.02), TAC (*p* = 0.04), and NO_3_^−^/NO_2_^−^ (*p* = 0.01) and decreased the LPO (*p* = 0.02) in the heart of MS rats undergoing ischemia/reperfusion. The results suggest that the treatment with an infusion from HSL calices protects the cardiac function from damage by ischemia and reperfusion through the antioxidant activities of the substances it possesses. It favors antioxidant enzymatic activities and nonenzymatic antioxidant capacity.

## 1. Introduction

Cardiovascular diseases (CVD) constitute one of the most prevalent health problems worldwide. These diseases have been strongly linked with metabolic syndrome (MS) which constitutes a complex condition associated with pathologies such as high blood pressure (BP), hypertriglyceridemia (TG), obesity, hyperinsulinemia, insulin resistance (IR), and oxidative stress (OS). OS is caused by a loss of the balance between the generation of reactive oxygen species (ROS) [1] and the capacity of the systems having antioxidant properties to neutralize ROS or to repair the resulting damage. OS is the result of an imbalance of the cellular redox state [2]. Furthermore, ROS excess has been involved in CVD, including aortic dilatation, aortic dissection, cardiac arrhythmias, coronary heart disease, left ventricular hypertrophy, and congestive heart failure [3].

Several experimental models in animals have been used to understand the participation of ROS in MS-associated CVD [4]. In our laboratory, we have studied a MS rat model that consumes chronically 30% sucrose in drinking water. In this model, glucose metabolism and the energy transfer in the heart are altered and CVD are favored [5]. Experiments in the isolated rat heart from male MS rats subjected to ischemia/reperfusion showed the development of lethal arrhythmias. Furthermore, the addition of insulin to the perfusion liquid administered to these hearts affected their mechanical work [6]. Other authors have reported that a high-sucrose diet for 2.5 weeks favored the development of early abnormalities of diastolic function followed by alterations in myocardial structure and systolic dysfunction 10 weeks after the end of the treatment [7]. Rats treated with 8% sucrose showed hypertension and tachycardia after 2 weeks of treatment [8]. In this last model, the cardiomyocytes were more susceptible to damage by oxidation since their antioxidant capacity was lower than their oxidative capacity, when compared to other cells. The OS present in the heart of the MS rats is characterized by the decrease in the activities of the enzymes SOD, CAT, and GPx [9]. The mitochondria of the heart of MS rats are decoupled, increasing the production of ROS, and this condition alters cardiac function [10].

Moreover, many studies have reported that the intake of the antioxidants contained in some foods contributes in diminishing the oxidation processes at the endogenous level, thus reducing the negative consequences derived from OS in diverse organs. *Hibiscus sabdariffa Linnaeus* (HSL) is known as aleluya in Cuba, meśta in India, and jamaica flower in México. HSL has been used to treat liver diseases, hypercholesterolemia, hypertriglyceridemia, gastrointestinal disorders, and hypertension [11]. The HSL calyces have compounds such as protocatechuic acid (PCA), anthocyanins, quercetin, catechins, and polyphenols that protect cellular components from damage by oxidation [12], decreasing lipid peroxidation (LPO), increasing the activities of CAT, GPx, and SOD [13], and participating in the regeneration of other antioxidants (vitamins C and E). It increases the GSH concentration and inhibits the xanthine oxidase (XO) activity and the angiotensin-converting enzyme (ACE). It also has an anti-inflammatory effect by modulating cyclooxygenase 2 and inducible nitric oxide (iNOS) synthase [14] and avoiding the prostaglandin E_2_ and nitric oxide (NO) synthesis [15]. It prevents liver cell apoptosis by inhibiting the activation of p-JNK and p38 MAPK transcription factors. It promotes low-serum TG and low-lipoprotein level density during inflammation, and it increases high-density lipoproteins, inhibiting the development and/or progression of atherosclerosis [16]. The perfusion using 12 polyphenols obtained from of HSL at doses ranging from 125 to 500 *μ*g/mL diminished systolic function in a recent work determining cardiac function in Langendorff preparations of rat hearts. The authors found negative inotropic, negative chronotropic, and positive lusitropic effects. The effect was attributed to calcium entry, its release, and reuptake [17], but the authors did not study the effect of these polyphenols on oxidative stress reduction and its implications for improving heart function. In addition, the HSL extract reverts fibrosis, inflammation, and hypertrophy and prevents heart failure in a rat model of myocardial infarction by isoprenaline [18]. Likewise, the chronic administration of an aqueous extract of HSL significantly attenuated and reversed cardiac hypertrophy in 2K-1C hypertensive rats through negative inotropic and chronotropic effects. This model is characterized by vascular ROS production. The protective mechanism was associated with the high concentrations of anthocyanins and vitamin C contained in the extract [19].

Here, we hypothesized that HSL infusion might have a positive effect reducing OS and thus prevent abnormalities in the MS rat heart. Therefore, the objective of this study was to evaluate myocardial protection from damage by ischemia/reperfusion due to the antioxidant effect of 3% HSL infusion in a MS rat model.

## 2. Material and Methods

### 2.1. Infusion

The HSL calyces were acquired in Chilapa de Alvarez (high zone in Guerrero, México). For the infusion preparation, 30 g of HSL calyces was incubated in a liter of boiling drinking water (95–100°C) for 10 min. The solution was then left to cool. It was filtered and 300 g sucrose was added. It was stored at 4°C until consumption. To determine total anthocyanin content of the infusion, 100 *μ* L was added to 50 mL of buffers (NaC_2_H_3_O_2_, 4 M) at pH 1 and 4.5, and the absorbance was measured at 520 and 700 nm and compared against a blank cell, filled with distilled H_2_O. The difference in the absorbance was used for calculating the cyanidin-3-glucoside (total monomeric anthocyanin) as described by the method of Lee et al. [20]. Total flavonoid content was determined by the method of Zhishen et al. [21]. In brief, 100 *μ*L of HSL infusion was added to 2175 *μ*L of distilled H_2_O plus 75 *μ*L of 5% NaNO_2_ and incubated for 3 min. Then, 150 *μ*L of 10% AlCl_3_ was added and the solution was incubated for 5 min. 0.5 mL of 1 M NaOH was added to the mixture, and it was shaken vigorously in a vortex. The absorbance was measured at 510 nm. The calibration curve was done using quercetin as standard. Total estimation of vitamin C was determined by the method of Jagota and Dani [22]. In brief, 100 *μ*L of the HSL infusion was added to 200 *μ*L of 0.20 mM Folin-Ciocalteu reagent. The mixture was shaken vigorously in a vortex for 5 seconds and incubated for 10 min. The absorbance was measured at 760 nm. The calibration curve was obtained using an ascorbic acid standard solution. The 3% HSL infusion contained 136.50 ± 58.50 mg/L of cyanidin-3-glucoside, 18.37 ± 0.48 mg/L of quercetin, and 1.45 ± 0.03 mM of vitamin C.

### 2.2. Animals

The Laboratory Animal Care Committee of the National Institute of Cardiology “Ignacio Chávez” in Mexico approved the experimental design, and experiments were done in accordance with the Guide for the Care and Use of Laboratory Animals of NIH. Weanling male rats weighing 200-250 g were used, *n* = 6 per group. The groups were as follows: control (Ctr), 30% sucrose fed (MS)—sucrose was added in their drinking water, and 30% sucrose fed plus HSL extract (MS + HSL) at the concentration of 30 g/L. The animals were placed in plastic boxes and were kept under 12 h light/obscurity cycles and environmental temperature ranging from 18 to 26°C. They were fed commercial rodent pellets (PMI Nutrition International Inc., LabDiet 5008, Richmond, IN) ad libitum. Weight of the rats and determinations of blood pressure (BP) were taken at the end of the experimental period of 12 weeks. BP was determined using a tail cuff attached to a pneumatic pulse transducer method (Narco Bio-Systems Inc., Houston, TX, USA), in compliance with the method described by Pérez-Torres et al. [23].

### 2.3. Isolated Heart Perfused by the Langendorff Method

The animals were anesthetized and given anticoagulants. After a thoracotomy, the heart was exposed, and with the help of a silk thread, the ascending aorta was referred. The heart was removed, placed in isotonic saline at 4°C, and connected to the perfusion system through the ascending aorta as previously described [24]. After an adaptation period of 30 min, the experimental perfusion conditions were established as previously described [25]. The determinations of left intraventricular pressure (LIVP), perfusion pressure (PP), and the values of HR (heart rate) and left intraventricular pressure (LIVP), cardiac mechanical performance (CMP), and coronary vascular resistance (CVR) were done as previously reported [25]. After, at the end of the experiments in the isolated perfused heart, the organ was stored at -30°C. It was homogenized in 0.25 mM sucrose solution, and total proteins were determined by the Bradford method as previously published [26]. The retroperitoneal fat tissue was also dissected and weighed. Blood samples were centrifuged for 20 min at 936 g and 4°C, in order to collect the serum in aliquots of 400 *μ*L and stored at -30°C for quantification of biochemical variables.

### 2.4. Mn and Cu/Zn Superoxide Dismutase and Peroxidase Activities

The activities of SOD enzyme isoforms were determined in the homogenate of the heart by nondenaturing gel electrophoresis and nitro blue tetrazolium (NBT) staining as described by Pérez-Torres et al. [23]. The NBT stain for O_2_ was viewed by UV light exposure for another 10 min. Riboflavin and TEMED in the presence of UV light and oxygen produce ROS; NBT and SOD compete with them. Where SOD is present, the gel remains transparent, whereas reduced NBT turns it purple-blue.

For the peroxidase activity, 35 *μ*L of horseradish peroxidase was loaded to a final concentration of 178.5 *μ*g as standard and 100 *μ*g of protein in the same conditions of the native gel as previously described. To observe the activity of the peroxidases, the gel was washed with distilled water three times, during 5 minutes, and it was then incubated with a mixture of 0.003 mg/mL 3,3′,5,5′-tetramethylbenzidine dissolved in a solution of ethanol : acetic acid : water (1 : 1 : 1 *v*/*v*) with H_2_O_2_ for 10 minutes in the dark. In these conditions, where peroxidases are present, the gel remains transparent and 3,3′,5,5′-tetramethylbenzidine is oxidized showing a green coloration. The gels for SOD isoform and peroxidase activities were analyzed by densitometry with the Kodak Image® 3.5, and activities were calculated following the technique described by Pérez-Torres et al. [23].

### 2.5. Glutathione Peroxidase

GPx activity was determined by a previously reported technique [27]. Activity is expressed in *μ*mol of NADPH oxidized/min/mg protein.

### 2.6. Glutathione S-Transferase

The activity of GST was determined spectrophotometrically in 100 *μ*g of protein, according the technique described by Beutler [28]. The sample was incubated and monitored for 10 min at 37°C at 340 nm. Values of GST activity were expressed in U/min/mg of protein. A unit of activity of GST is expressed in *μ*mol of GS-DNB conjugate formed/min/mg of protein at 37°C.

### 2.7. Glutathione Reductase

The GR activity was determined according to the method described by Agarkov et al. [29]. The absorbance was read at 340 nm. GR activity is expressed in U/min/mL of protein.

### 2.8. GSH Concentration

The GSH concentration in 100 *μ*g of protein of heart homogenate was made according to a previous method by Ellman [30], and absorbance was read at 412 nm. The calibration curve was done with GSH at concentrations from 5 to 25 *μ*mol.

### 2.9. Lipid Peroxidation (LPO)

LPO was determined by a previously reported technique [23], after extracting the products to an n-butanol phase and measuring the absorbance at 532 nm. The calibration curve was obtained using tetraethoxypropane as standard.

### 2.10. Evaluation of Total Antioxidant Capacity (TAC)

The total antioxidant capacity was measured by a previously reported technique [31]. The calibration curve was obtained using Trolox.

### 2.11. Nitrate and Nitrite Ratio Quantification

NO_3_^−^ was reduced to NO_2_^−^ by the Cu-Cd reaction as previously reported [23]. The calibration curve was obtained with KNO_2_ solution of 5–0.156 nM. The absorbance was measured at 540 nm.

### 2.12. Biochemical Variables

Commercially obtained ELISA kits were used for the determination of some serum biochemical variables from the rats, such as glucose, cholesterol, TG, and insulin. The HOMA index of resistance to insulin was calculated. HOMA − IR = (insulin *μ*U/mL)^∗^ (glucose mM/L)/22.5.

### 2.13. Histological Preparation

The histological sections of the left ventricle were done after the ventricle had been washed in 0.9% NaCl for 30 sec. and fixed by immersion in phosphate buffer with 10% formalin (pH 7.4) for 24 h. The sections were processed according to conventional histological procedures by hematoxylin-eosin stain. A Carl Zeiss light microscope (Carl Zeiss Axio Imager Z2, West Germany) with objective EC Plan-Neofluar 10x, with an HP Z800 computer and HP ZR30W screen, was used to analyze the histological sections. The photomicrographs were studied using densitometry with the SigmaScan Pro 5 Image Analysis software. The density values are expressed as pixel units.

### 2.14. Statistical Analysis

Statistical analysis and graphs were performed with a SigmaPlot 12.3 program (version 2016, Systat Software Inc., San Jose, CA, USA). The data are presented as the mean ± SE. Statistical significance was determined by one-way ANOVA test, followed by the post-hoc Tukey test. Differences were considered statistically significant when *p* < 0.05.

## 3. Results

### 3.1. General Variables

Table 1 shows general characteristics of the experimental animals. The insulin, HOMA-index, TG, intra-abdominal fat, and BP were significantly elevated in MS in comparison with Ctr (*p* = 0.001). The administration of HSL reduced these variables in comparison to MS (*p* < 0.04). Cholesterol and glucose remained at normal levels in all groups.

### 3.2. Perfusion of the Isolated Heart

**Figure 1:**
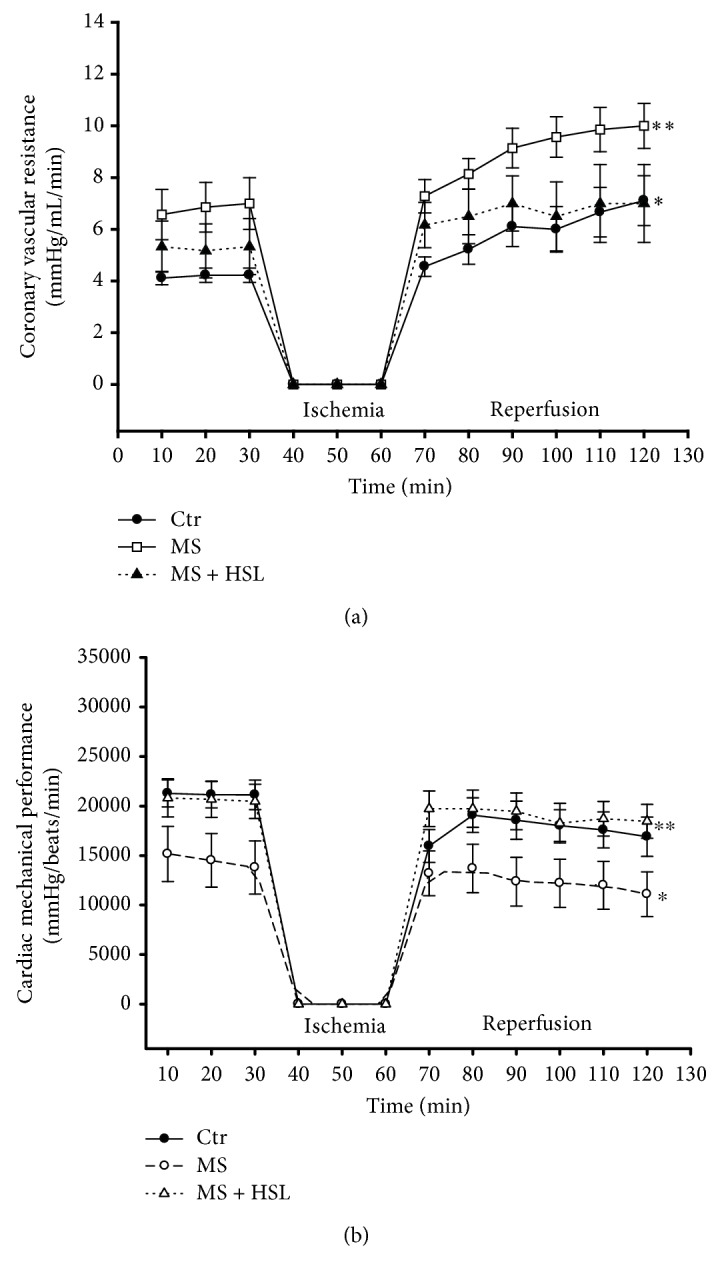
Isolated perfused heart in experimental rats. (a) Coronary vascular resistance of hearts with global ischemia (30 min) and reperfusion (60 min) periods (*n* = 6); ^∗^*p* = 0.01 Ctr vs. MS and ^∗∗^*p* = 0.04 MS vs. MS + HSL. (b) Cardiac mechanical performance of isolated hearts with global ischemia (30 min) and reperfusion (60 min) (*n* = 6); ^∗^*p* = 0.03 Ctr vs. MS and ^∗∗^*p* = 0.01 MS vs. MS + HSL. Ctr: control; MS: metabolic syndrome; MS + HSL: metabolic syndrome plus *Hibiscus sabdariffa Linnaeus.*

Figure 1(a) describes the coronary vascular resistance (CVR) in the experimental groups. In the Ctr group, a CVR of 4 mmHg/mL/min in the initial period was found and it increased 60% at the end of the reperfusion period. These variations are normal under this condition. The MS group had a 50% increase in CVR in comparison with the Ctr group in the initial period, and it increased 2-fold in the reperfusion period showing a statistically significant difference (*p* = 0.01). After the treatment with the HSL infusion, CVR was restored in MS rats (*p* = 0.04) and it was in a value similar to the one found in Ctr rats.  Figure 1(b) shows that the CMP in Ctr hearts is decreased by 20% at the end of the reperfusion period. This diminution reflects the damage generated by the global ischemia, in addition to the damage caused by reperfusion. The same behavior was observed in MS + HSL hearts. In comparison, MS hearts without treatment had a CMP that was decreased by 50% when compared to the Ctr and SM + HSL groups (*p* = 0.03 and *p* = 0.01, respectively).

### 3.3. SOD Isoform Activities

**Figure 2:**
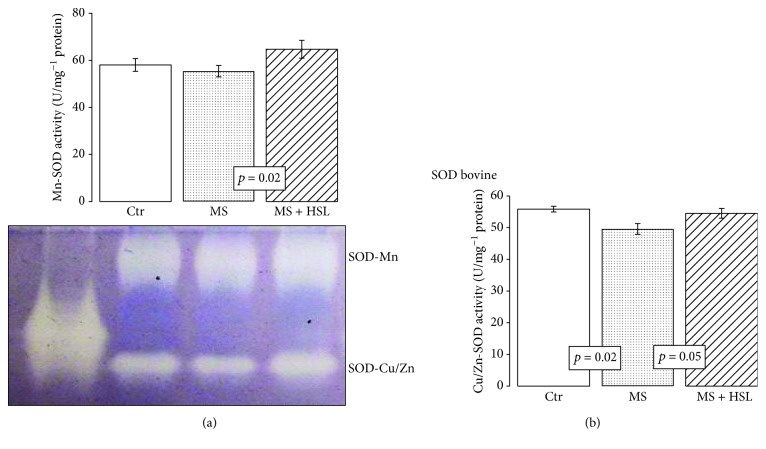
Densitophotometric analysis of activity of super oxide dismutase isoforms in the heart homogenates of the experimental rats; a native gel representative of the Mn-SOD and Cu/Zn-SOD activities is presented. (a) Manganese isoform and (b) copper/zinc isoform. Native gel electrophoresis with 10% polyacrylamide. Ctr: control; MS: metabolic syndrome; MS + HSL: metabolic syndrome plus *Hibiscus sabdariffa Linnaeus.* Data are expressed in mean ± SE (*n* = 6 rats in each group).

In the heart homogenate of the MS rats, the Mn-SOD activity was not significantly modified when compared to that found in Ctr rats. However, the HSL treatment significantly increased the activity of these isoforms in MS rats (*p* = 0.02, Figure 2(a)). The Cu/Zn-SOD activity in heart homogenate from the MS rats showed a significant decrease in comparison to the activity found in Ctr and MS + HSL rat hearts (*p* = 0.02, *p* = 0.05, respectively, Figure 2(b)).

### 3.4. Glutathione-Dependent Enzymes in the Heart

**Figure 3:**
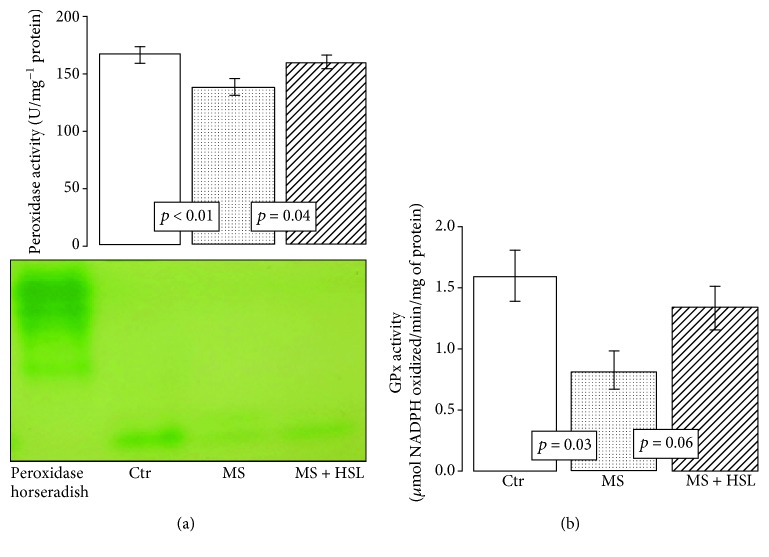
(a) Densitophotometric analysis of peroxidase activities. A native gel electrophoresis with 10% polyacrylamide is shown between (a) and (b). Under these conditions, where peroxidases are present, the gel remains transparent and the 3, 3′,5,5′-tetramethylbenzidine is oxidized showing a green coloration. (b) GPx activity has a tendency to increase its activity when comparing MS + HSL vs. MS. However, the change was not statistically significant (*p* = 0.06 NS). Ctr: control; MS: metabolic syndrome; MS + HSL: metabolic syndrome plus *Hibiscus sabdariffa Linnaeus*. Data are expressed in mean ± SE (*n* = 6 rats in each group).

**Figure 4:**
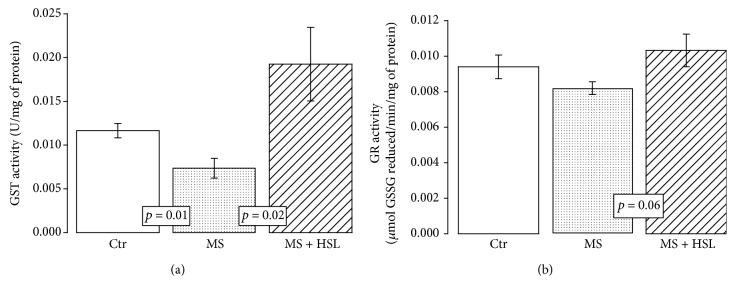
(a) Glutathione S-transferase activity in the experimental groups and (b) effect of the treatment with HSL infusion on glutathione reductase activity in the heart homogenate. There was a tendency to an increase in the activity of GR between MS + HSL and MS; however, it did not reach a statistically significant level (*p* = 0.06 NS). Ctr: control; MS: metabolic syndrome; MS + HSL: metabolic syndrome plus *Hibiscus sabdariffa Linnaeus*. Data are expressed in mean ± SE (*n* = 6 rats in each group).

Figure 3(a) shows that the activity of the peroxidases in the heart homogenates was significantly decreased in MS rats in comparison with Ctr and MS + HSL rats (*p* < 0.01 and *p* = 0.04, respectively).  Figure 3(b) shows that the activity of GPx was significantly decreased in the MS group when compared to the Ctr group (*p* = 0.03). However, the treatment with HSL in MS rats only showed a tendency to increase its activity without reaching a significant change (*p* = 0.06).  Figure 4(a) shows that the activity of GST was significantly decreased in the MS group in comparison to the Ctr group (*p* = 0.01). The treatment with HSL in MS rats significantly increased the GST activity (*p* = 0.02). The enzymatic activity of the GR was not significantly increased in the MS vs. the Ctr group. When the activity of the enzyme was compared with that from the MS + HSL group, it only showed a tendency to increase without reaching a statistically significant change (*p* = 0.06, Figure 4(b)).

### 3.5. GSH Levels

**Figure 5:**
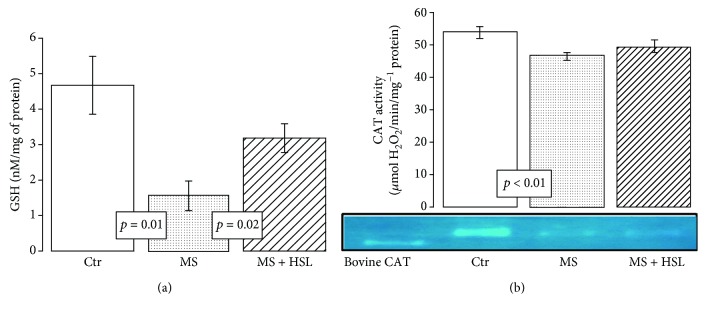
Effect of treatment with HSL infusion. (a) Reduced GSH concentrations. (b) Densitophotometric analyses of CAT activity in the heart homogenate. In (b), a native representative gel of the CAT activity is shown. Ctr: control; MS: metabolic syndrome; MS + HSL: metabolic syndrome plus *Hibiscus sabdariffa Linnaeus*. Data are expressed in mean ± SE (*n* = 6 rats in each group).

Figure 5(a) shows that the GSH in the heart homogenate was significantly diminished in the MS group when compared to the Ctr group (*p* = 0.01). The treatment with the HSL infusion in MS rats significantly increased the GSH levels (*p* = 0.02).

### 3.6. Catalase Activity

The MS rats showed a significant decrease in CAT activity in comparison to Ctr rats (*p* < 0.001). The HSL infusion did not significantly modify the CAT activity in the MS rats (Figure 5(b)).

### 3.7. Oxidative Markers in Heart Homogenate

**Figure 6:**
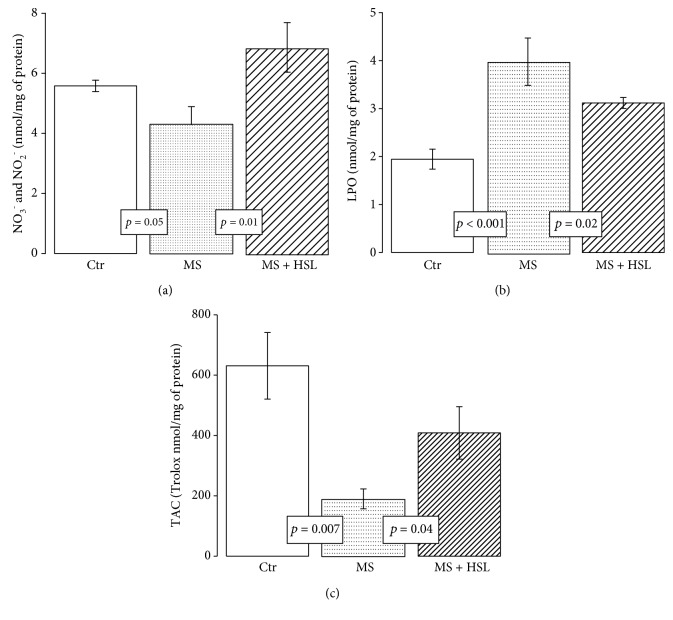
(a) Nitrate and nitrite ratio in the heart homogenates from the experimental groups. (b) Lipid peroxidation levels and (c) total antioxidant capacity. Ctr: control; MS: metabolic syndrome; MS + HSL: metabolic syndrome plus *Hibiscus sabdariffa Linnaeus*. Data are expressed in mean ± SE (*n* = 6 rats in each group).

The NO_3_^−^/NO_2_^−^ ratio was significantly decreased in MS rats in comparison with Ctr and MS + HSL rats (*p* = 0.05 and *p* = 0.01, respectively, Figure 6(a)). Regarding LPO, which is a LPO marker, the MS group showed a significant increase in its levels in comparison to the Ctr and MS + HSL groups (*p* < 0.001 and *p* = 0.02, respectively, Figure 6(b)). In addition, Figure 6(c) shows that the TAC in the heart homogenate was decreased in MS when compared to its level in Ctr rats (*p* = 0.007). The HSL treatment significantly increased its level (*p* = 0.04).

### 3.8. Heart Histology

**Figure 7:**
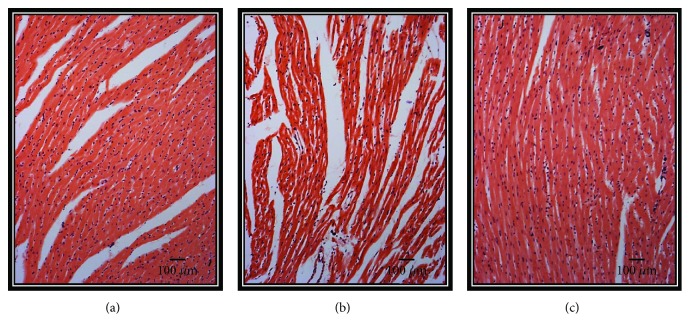
(a–c) Representative photomicrographs of heart tissue after of reperfusion from the three experimental groups. 5 fields per sample were analyzed. (a) Ctr, (b) MS, and (c) MS + HSL. Values are the mean ± SE (*n* = 6). The tissue was processed according to conventional histological procedures, and histological sections were made and stained by hematoxylin-eosin stain at 10x. Ctr: control; MS: metabolic syndrome; MS + HSL: metabolic syndrome plus *Hibiscus sabdariffa Linnaeus.*

Figure 7(a) shows the section of a myocardium from the Ctr group in which contraction bands are arranged in compact bundles of myocytes, separated by fibrous bands. It is possible to distinguish focally ovoid nuclei and intercalated disks, as part of the normal histologic aspect of the myocardium. In the hearts from the MS group, there are slight changes in relation to the Ctr group. A variable undulation in the bundles of myofibrils is observed, and it is possible to point out that they are narrower than those in the controls in the longitudinal section (Figure 7(b)). There is also edema between them, loss of striations, and focally incipient necrosis. We did not find hemorrhage, hypereosinophilia, or inflammatory cells or polymorph nuclear leukocytes. In the HSL-treated hearts, there were clearly less changes in the morphology than those found in the MS group. The hearts from this group showed similar characteristics to those found in the Ctr group (Figure 7(c)). All representative microphotographs of the Ctr, MS, and MS + HSL groups were taken from areas irrigated by the left anterior descending coronary artery, from the tip of the heart and from the anterior wall of the left ventricle and approximately two-thirds anterior to the ventricular septum.

**Figure 8:**
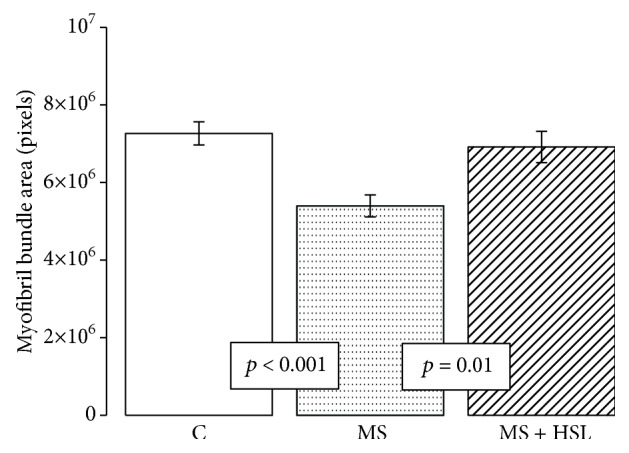
Densitophotometric analysis of myocyte bundle areas in experimental groups. Ctr: control; MS: metabolic syndrome; MS + HSL: metabolic syndrome plus *Hibiscus sabdariffa Linnaeus.* Values are the mean ± SE (*n* = 6).

Figure 8 describes the densito-photometric analysis of myocyte bundle areas which showed a significant decrease in the myocardium from the MS group in comparison to the Ctr and MS + HSL groups (*p* < 0.001 and *p* = 0.01, respectively).

## 4. Discussion

MS is a cluster of pathologies that includes hyperlipidemia, hypertension, obesity, and IR and is considered as risk factors for CVD [32]. Cardiomyopathy in the MS is characterized by complex changes in the mechanical, biochemical, structural, and electrical properties of the heart. In both MS and CVD, the redox equilibrium is altered towards OS [33]. Traditional medicine has allowed people to identify several nutrients, food supplements, herbs, and spices which exhibit anti-MS and antiobesity effects [34]. HSL is one of the medicinal herbs commonly used in traditional Asian and African medicine against hypertension, obesity, and hypercholesterolemia. It has been described that a crude extract of HSL exhibits antihypertensive and cardioprotective effects on hypertensive rats [35]. The aim of this study was to evaluate the myocardial protection induced by a 3% infusion of HSL against ischemia-reperfusion damage due to its antioxidant effect on a MS rat model.

### 4.1. Isolated Perfused Heart and Hypertension

Our results show that there are altered CVR, CMP, and anatomical changes in the hearts from MS rats after an ischemic insult and that reperfusion further alters this condition. These changes have been previously reported [6]. In the MS model induced by a high-sucrose diet, there is decreased mitochondrial function that contributes to a diminution of the ATP levels reaching the myofibrils, leading to a decrease in CMP [5]. In this paper, we found that the treatment with an HSL infusion improves CMP and CVR, by decreasing the ROS exacerbation. It has been reported that HSL infusion might also act on the heart by promoting a Ca^2+^ flow. Both of these actions, reduction of OS and Ca^2+^ flow, contribute to negative inotropic and chronotropic effects.

Anthocyanins such as delphinidin and cyanidin-3-0-sambubiosides are present in HSL and may block Ca^2+^ channels in vascular smooth muscle, reducing vasoconstriction [36]. It has also been reported that HSL polyphenols ameliorate cardiac dysfunction and vasodilatation, via modulation of intracellular Ca^2+^ entry and reuptake in the heart [17]. Another report showed that oral consumption of HSL enhanced cardiac Na^+^-K^+^-ATPase and Ca^2+^-Mg^2+^-ATPase activities in a rat model of hypertension [37]. The finding of vasorelaxation in isolated rat coronary arteries indicated that there is a Ca^2+^ inhibitory action by flavonoids such as quercetin via the inhibition of extracellular Ca^2+^ influx-induced contraction, reduced intracellular-free Ca^2+^ concentration, and an inhibition of the inward Ca^2+^ currents through voltage-dependent Ca^2+^ channels [38].

Other metabolic pathways have been proposed to explain the beneficial effects of HSL on the heart. A recent study reported both negative inotropic and chronotropic actions of HSL aqueous extract in isolated atria and attributed this action to hibiscus acid, the main phenolic acid present in HSL [39], and to the polyphenol and resveratrol present in the HSL infusion. These compounds reversed remodeling and improved inotropic function in a rodent model of heart failure [40]. Another study reported a beneficial effect of the HSL extract in isolated hearts indicating that the extract modulates negative inotropic effects through the antagonistic action on the *β*-adrenergic receptor [41]. In this model, a significant increase in the vasoconstriction in aortic rings by norepinephrine was associated to damage in the endothelium. The HSL treatment decreased this damage, and this was associated with low BP [13].

In addition, another study showed that HSL effectively ameliorated the systolic dysfunction of the heart. This was evidenced by a significant rise in the development of left ventricular pressure and in cardiomyocyte hypertrophy. The HSL extract reduced gene expression of the myocyte hypertrophic machinery and contributed to a reduction of cardiac fibrosis improving ventricular compliance and relaxation. This was associated with a decrease of OS [42]. The above finding suggests that the inotropic and chronotropic actions by HSL infusion in the heart of the MS rats can contribute to restored CMP and CVR. This would be partly reflected in the BP decrease in MS rats. In turn, this may also favor cardiac mechanics.

Polyphenols present in HSL activate the PI3 K pathway in the endothelium. This pathway is capable of upregulating nitric oxide (NO) via phosphorylation of eNOS. It also suppresses spontaneous Ca^2+^ events in isolated cardiac myocytes and in the isolated rat heart thus decreasing cardiac arrhythmias [17]. Furthermore, the NO synthesized by this pathway can increase endothelial vasorelaxation and lead to a decrease and/or increase in BP and in the NO^3-^/NO^2-^ ratio, which is an index of NO release. This was shown by our results where there was a normalization of the cardiac function and BP.

Herrera-Arellano et al. have demonstrated in a clinical trial that in patients with stage I and II hypertension, the HSL extract has antihypertensive action. Promotion of diuresis or ACE inhibition could underlie this antihypertensive action [43]. When HSL is ingested for a long term, there is heart hypertrophy in the spontaneously hypertensive rats and a decrease in BP and left ventricular mass. There is also an increase in the surface area and in the length and density of myocardial capillaries [44]. In addition, the anthocyanins delphinidin and cyanidin-3-0-sambubiosides present in HSL have an antihypertensive effect thanks to two synergistic and complementary mechanisms of action: the first is by acting as diuretics, probably due to pharmacokinetics similar to those of aldosterone antagonists (increases the elimination of water and natriuresis without modification in the excretion of potassium), and the second is by competition with the ACE [43].

### 4.2. Antioxidant Enzymes: SOD, CAT, GPx, and Peroxidases

During ischemia and reperfusion, the myocardium produces ROS which play a major role in the reperfusion injury. ROS production causes Ca^2+^ reentry, infiltration by inflammatory cells, platelet activation, NO production, metabolic alterations, and endothelial dysfunction. These changes contribute to the background of OS that characterizes the MS model [23]. The activity of antioxidant enzymes is regulated according to cellular requirements, since their production can be induced or inhibited by endogenous effectors. Among the main antioxidant enzymes are SOD isoforms. There are three isoforms of this enzyme, Cu/Zn-SOD located in the cytoplasm, Mn-SOD that is present in the mitochondrial matrix, and EC-SOD located in plasma [45]. An elevation of SOD isoforms by HSL treatment may contribute to reduce superoxide (O_2_^−^) in the heart of the MS rats, and this can in part help improve cardiac function. However, this leads to an increase in H_2_O_2_. To detoxify this peroxide, cardiomyocytes have two systems. The first is CAT which is a hemoprotein with four heme groups, located in peroxisomes and mitochondria. This enzyme is responsible for detoxifying H_2_O_2_, following a concentration gradient and converting it into H_2_O and molecular O_2_ [46]. The HSL treatment did not modify this enzyme's activity; its levels were low and contributed to OS and to the deterioration of cardiac function. However, this may be due to the overproduction of H_2_O_2_. When this substrate is used by CAT, it inhibits its activity. However, the PGx enzyme selenoprotein found in the mitochondrial matrix and in the cytoplasm can also detoxify the H_2_O_2_ and other organic hydroperoxides converting them into H_2_O and molecular O_2_. This enzyme does not depend on the concentration gradient being dependent on the presence of NADPH^+^ and GSH [47]. The increased tendency of the GPx and elevated peroxidase activities by HSL treatment suggest that HSL treatment can modulate the activity of these enzymes, favoring an increase in their activity, thus contributing to the decrease of chronic OS in the heart. Previous studies showed that the HSL extract significantly increased GPx and SOD in *Cyprinus carpio* hepatocytes by carbon tetrachloride toxicity [48]. Also, HSL treatment overexpressed the GPx in renal ischemia-reperfusion injury in mice by acetaminophen toxicity [49]. In addition, the extract of *Hibiscus rosa-sinensis*, another flower of the *Malvaceae* family, significantly raised GSH levels and SOD and CAT activities and decreased LPO in myocardial ischemia/reperfusion in rats [50]. In addition, GPx catalyzes the reduction of H_2_O_2_. To carry out its antioxidant activity, GPx requires GSH, which is a critical molecule in the defense against OS that maintains the reducing environment of the cardiomyocytes producing its oxidized form GSSG. The GR reverts this substrate to its reduced form [51].

### 4.3. Glutathione and GR Activity

GSH is the most versatile antioxidant because of its variety of functions that include the detoxification of xenobiotics or their metabolites. It is the largest source of endogenous antioxidants, and it inhibits the radicals -OH and O_2_. It also regenerates vitamins E and C, reconverting them to their active form, and it acts as a cofactor for the GPx enzymes. Approximately 90% of the GSH present in the cardiomyocytes is found in the cytoplasm, while 10% is located in the mitochondria. Eighty-five percent of the total cellular GSH is free, and the rest is bound to proteins. It is transported by amino acids through the plasma membrane acting as a storage source of cysteine [52]. GSH raises and, together with the tendency to increase the GR activity, suggests that HSL infusion may contribute to GSH increase through its regeneration by the GR activity in the heart of the MS rats. The HSL treatment may also contribute the GSH increase through the PCA present in the HSL calyces, what attenuates the OS [53]. In addition, another study showed that the polyphenol extract of HSL increases GSH in the damaged liver by acetaminophen [54]. However, the GSH concentration not only comes from this metabolic pathway but also depends on its precursor amino acids cysteine, glutamate, and glycine or on a decrease/increase in the activity of the enzymes that synthesize it such as *γ*-glutamyl-cysteine synthetase and GSH synthetase [47].

### 4.4. Glutathione S-Transferase Activity, TAC, and LPO

On the other hand, GST is the enzyme involved in xenobiotic metabolism and in the protection of damage caused by peroxidated lipids. It catalyzes the GSH ionization to the form of a nucleophilic thiolate anion which reacts spontaneously with nucleophilic components that are closely located. This reaction is followed by the conjugation of the substrate, the formation of the product, and its release. The conjugation increases the solubility of the toxic products, facilitating their excretion from the cell [55], and decreases the LPO rate, which may cause toxicity. HSL treatment increases GST activity and TAC while decreasing LPO. This suggests that the decreased GST activity in the heart of the MS rats favors OS, which is evidenced by the LPO increase. The antioxidant properties of the HSL infusion can contribute in raising GST activity associated with the decrease in LPO. This, in turn, increases the TAC. A recent study in the aortic aneurysm of Marfan syndrome patients showed that an infusion of 2% HSL increased GST activity and TAC and this was associated with LPO decrease [56]. In addition, another study showed a decrease in GST activity related to an increase in ROS in hypertension [57]. In the same way, it has been described that GSTA4 expression is downregulated in the adipose tissue from obese insulin-resistant C57BL/6 J mice and in humans with obesity-linked IR [58]. This model is characterized by IR and hyperinsulinemia which can contribute in inhibiting the GST activity, increasing the LPO, and decreasing the TAC in the heart of MS rats. Furthermore, it has been described that IR and hyperinsulinemia lead to structural abnormalities in the heart, such as increased left atrial size and left ventricular mass [59]. Also, the treatment of HSL infusion decreases the HOMA index; this effect may be due to the presence of cyanidin-3-glucoside which can over express the GLUT4 transporter and can increase the signaling of insulin by the cells [60]. Moreover, *in vitro* studies have shown that the polyphenolic fraction from the aqueous HSL extracts increases the TAC [55]. This may be because the HSL polyphenols participate as captors of ROS in a second line of defense when they have not been neutralized by the enzymatic antioxidant system [32]. They also increase the antioxidant capacity of the nonenzymatic system, favoring an increase of TAC in the heart of MS rats. In the H9c2 cardiomyoblast cells, the protective effect of HSL on doxorubicin-induced cytotoxicity was through the attenuation of ROS production and by inhibition of xanthine oxidase activity, by the reduction of LPO and by the elevation of the antioxidant enzyme activity. Therefore, TAC was observed [61]. In addition, the hydrophilic antioxidants present in HSL have scavenging properties through which they are able to inhibit the free radical mechanism of LPO [62].

On the other hand, the decrease in serum TG by the HSL treatment could be explained by the amount of soluble fiber present in the calices of the HSL plant. It has been estimated that each 250 mL of HSL infusion contains 166 mg of fiber [11].

## 5. Conclusion

Based on these results, it can be concluded that treatment with HSL infusion protects the cardiac function during ischemia and reperfusion through the action of the antioxidant substances that it possesses such as PCA, anthocyanins, cyanidin-3-glucoside, quercetin, and polyphenols, thus favoring antioxidant enzymatic activities and nonenzymatic antioxidant capacity.

## Figures and Tables

**Table 1:** General characteristic of experimental groups.

Variables	Ctr	MS	MS + HSL
Glucose (mmol/L^−1^)	114.6 ± 4.3	115.3 ± 3.5	105.1 ± 6.9
Insulin (*μ*U/mL^−1^)	4.8 ± 0.6	18.1±4.0^∗∗^	7.4 ± 1.0^∗^
HOMA index	1.3 ± 0.1	4.9±1.2^∗∗^	2.1 ± 0.3^∗^
Triglycerides (mg/dL^−1^)	56.1 ± 4.0	132.0±15.5^∗∗^	90.0 ± 11.4^∗^
Cholesterol (mg/dL^−1^)	43.4 ± 1.3	41.6 ± 1.7	43.6 ± 4.1
Intra-abdominal fat (g)	3.3 ± 0.3	10.8±1.3^∗∗^	6.4 ± 0.5^∗^
Systolic blood pressure (mmHg)	115.1 ± 0.2	138.1±0.8^∗∗^	128.1 ± 2.7^∗^
Body mass (g)	812.2 ± 18.7	821.1 ± 22.4	816.1 ± 16.3

Data are mean ± SE, *n* = 6 each group. Statistically significant at ^∗^*p* < 0.04 MS vs. MS + HSL; ^∗∗^*p* = 0.001 Ctr vs. MS. Ctr: control; MS: metabolic syndrome; MS + HSL: metabolic syndrome plus *Hibiscus sabdariffa Linnaeus*.

## Data Availability

The data used to support the findings of this study are available from the corresponding author upon request.

## References

[1] Dröge W. (2002). Free radicals in the physiological control of cell function. *Physiological Reviews*.

[2] Murdoch C. E., Zhang M., Cave A. C., Shah A. M. (2006). NADPH oxidase-dependent redox signalling in cardiac hypertrophy, remodelling and failure. *Cardiovascular Research*.

[3] Yue Y., Qin Q., Cohen M. V., Downey J. M., Critz S. D. (2002). The relative order of mKATP channels, free radicals and p38 MAPK in preconditioning’s protective pathway in rat heart. *Cardiovascular Research*.

[4] Halushka M. K. (2012). Single gene disorders of the aortic wall. *Cardiovascular Pathology*.

[5] Carvajal K., Baños G., Moreno-Sánchez R. (2003). Impairment of glucose metabolism and energy transfer in the rat heart. *Molecular and Cellular Biochemistry*.

[6] Cárdenas G., Torres J. C., Zamora J., Baños G. (2005). Isolated heart function during ischemia and reperfusion in sucrose-fed rats. Effect of insulin infusion. *Cardiovascular Pathology*.

[7] Vasanji Z., Cantor E. J. F., Juric D., Moyen M., Netticadan T. (2006). Alterations in cardiac contractile performance and sarcoplasmic reticulum function in sucrose-fed rats is associated with insulin resistance. *American Journal of Physiology-Cell Physiology*.

[8] Buñag R. D., Tomita T., Sasaki S. (1983). Chronic sucrose ingestion induces mild hypertension and tachycardia in rats. *Hypertension*.

[9] Baños G., Medina-Campos O. N., Maldonado P. D. (2005). Activities of antioxidant enzymes in two stages of pathology development in sucrose-fed rats. *Canadian Journal of Physiology and Pharmacology*.

[10] Carvajal K., El Hafidi M., Marin-Hernández A., Moreno-Sánchez R. (2005). Structural and functional changes in heart mitochondria from sucrose-fed hypertriglyceridemic rats. *Biochimica et Biophysica Acta*.

[11] Sáyago-Ayerdi S. G., Arranz S., Serrano J., Goñi I. (2007). Dietary fiber content and associated antioxidant compounds in Roselle flower (*Hibiscus sabdariffa* L.) beverage. *Journal of Agricultural and Food Chemistry*.

[12] Ali B. H., Mousa H. M., el-Mougy S. (2003). The effect of a water extract and anthocyanins of Hibiscus sabdariffa L. on paracetamol-induced hepatoxicity in rats. *Phytotherapy Research*.

[13] Pérez-Torres I., Zúñiga Muñoz A., Beltrán-Rodríguez U., Díaz-Díaz E., Martínez-Memije R., Guarner Lans V. (2013). Modification of the liver fatty acids by *Hibiscus sabdariffa* Linnaeus (Malvaceae) infusion, its possible effect on vascular reactivity in a metabolic syndrome model. *Clinical and Experimental Hypertension*.

[14] Chen C. C., Hsu J. D., Wang S. F. (2003). *Hibiscus sabdariffa* extract inhibits the development of atherosclerosis in cholesterol-fed rabbits. *Journal of Agricultural and Food Chemistry*.

[15] Wang S. C., Lee S. F., Wang C. J., Lee C. H., Lee W. C., Lee H. J. (2011). Aqueous extract from *Hibiscus sabdariffa* Linnaeus ameliorate diabetic nephropathy via regulating oxidative status and Akt/Bad/14-3-3*γ* in an experimental animal model. *Evidence-based Complementary and Alternative Medicine*.

[16] Willcox J. K., Ash S. L., Catignani G. L. (2004). Antioxidants and prevention of chronic disease. *Critical Reviews in Food Science and Nutrition*.

[17] Lim Y. C., Budin S. B., Othman F., Latip J., Zainalabidin S. (2017). Roselle polyphenols exert potent negative inotropic effects via modulation of intracellular calcium regulatory channels in isolated rat heart. *Cardiovascular Toxicology*.

[18] Ali S. S., Mohamed S. F. A., Rozalei N. H., Boon Y. W., Zainalabidin S. (2019). Anti-fibrotic actions of roselle extract in rat model of myocardial infarction. *Cardiovascular Toxicology*.

[19] Odigie I. P., Ettarh R. R., Adigun S. A. (2003). Chronic administration of aqueous extract of Hibiscus sabdariffa attenuates hypertension and reverses cardiac hypertrophy in 2K-1C hypertensive rats. *Journal of Ethnopharmacology*.

[20] Lee J., Durst R. W., Wrolstad R. E. (2005). Determination of total monomeric anthocyanin pigment content of fruit juices, beverages, natural colorants, and wines by the pH differential method: collaborative study. *Journal of AOAC International*.

[21] Zhishen J., Mengcheng T., Jianming W. (1999). The determination of flavonoid contents in mulberry and their scavenging effects on superoxide radicals. *Food Chemistry*.

[22] Jagota S. K., Dani H. M. (1982). A new colorimetric technique for the estimation of vitamin C using Folin phenol reagent. *Analytical Biochemistry*.

[23] Pérez-Torres I., Roque P., el Hafidi M., Diaz-Diaz E., Baños G. (2009). Association of renal damage and oxidative stress in a rat model of metabolic syndrome. Influence of gender. *Free Radical Research*.

[24] Li D., Li N. S., Chen Q. Q. (2008). Calcitonin gene-related peptide-mediated cardioprotection of postconditioning in isolated rat hearts. *Regulatory Peptides*.

[25] Döring H. J., Dehvert H., Dehnert H. (1988). The isolated perfused warm-blooded heart according to Langendorff. *Biological Measurement Techniques*.

[26] Bradford M. M. (1976). A rapid and sensitive method for the quantitation of microgram quantities of protein utilizing the principle of protein-dye binding. *Analytical Biochemistry*.

[27] Flohé L., Günzler W. A. (1984). [12] Assays of glutathione peroxidase. *Methods in Enzymology*.

[28] Beutler E. (1988). The relationship of red cell enzymes to red cell life-span. *Blood Cells*.

[29] Agarkov A. A., Popova T. N., Verevkin A. N., Matasova L. V. (2014). Activity of the glutathione antioxidant system and NADPH-generating enzymes in blood serum of rats with type 2 diabetes mellitus after administration of melatonin-correcting drugs. *Bulletin of Experimental Biology and Medicine*.

[30] Ellman G. L. (1959). Tissue sulfhydryl groups. *Archives of Biochemistry and Biophysics*.

[31] Benzie I. F. F., Strain J. J. (1996). The ferric reducing ability of plasma (FRAP) as a measure of ‘antioxidant power’: the FRAP assay. *Analytical Biochemistry*.

[32] Perez-Torres I., Ruiz-Ramirez A., Banos G., el-Hafidi M. (2013). Hibiscus sabdariffa Linnaeus (Malvaceae), curcumin and resveratrol as alternative medicinal agents against metabolic syndrome. *Cardiovascular & Hematological Agents in Medicinal Chemistry*.

[33] Zhang Y., Tocchetti C. G., Krieg T., Moens A. L. (2012). Oxidative and nitrosative stress in the maintenance of myocardial function. *Free Radical Biology and Medicine*.

[34] Aggarwal B. B. (2010). Targeting inflammation-induced obesity and metabolic diseases by curcumin and other nutraceuticals. *Annual Review of Nutrition*.

[35] Onyenekwe P. C., Ajani E. O., Ameh D. A., Gamaniel K. S. (1999). Antihypertensive effect of roselle (Hibiscus sabdariffa) calyx infusion in spontaneously hypertensive rats and a comparison of its toxicity with that in Wistar rats. *Cell Biochemistry and Function*.

[36] McKay D. L., Chen C. Y. O., Saltzman E., Blumberg J. B. (2010). *Hibiscus sabdariffa* L. tea (tisane) lowers blood pressure in prehypertensive and mildly hypertensive adults. *The Journal of Nutrition*.

[37] Olatunji L. A., Usman T. O., Adebayo J. O., Olatunji V. A. (2012). Effects of aqueous extract of Hibiscus sabdariffa on renal Na(+)-K(+)-ATPase and Ca(2+)-Mg(2+)-ATPase activities in Wistar rats. *Zhong Xi Yi Jie He Xue Bao*.

[38] Hou X., Liu Y., Niu L., Cui L., Zhang M. (2014). Enhancement of voltage-gated K^+^ channels and depression of voltage-gated Ca^2+^ channels are involved in quercetin-induced vasorelaxation in rat coronary artery. *Planta Medica*.

[39] Micucci M., Malaguti M., Gallina Toschi T. (2015). Cardiac and vascular synergic protective effect of *Olea europea* L. leaves and *Hibiscus sabdariffa* L. flower extracts. *Oxidative Medicine and Cellular Longevity*.

[40] Kanamori H., Takemura G., Goto K. (2013). Resveratrol reverses remodeling in hearts with large, old myocardial infarctions through enhanced autophagy-activating AMP kinase pathway. *The American Journal of Pathology*.

[41] Bako I. G., Abdulwahab A., Sudi A., Ikuku A. J., Abubakar M. S., Maje I. M. (2013). Effects of aqueous seed extract of Hibiscus sabdariffa linn (malvaceae) on isolated perfused rabbit heart. *International Journal of Medical Science and Biotechnology*.

[42] Si L. Y. N., Ali S. A. M., Latip J., Fauzi N. M., Budin S. B., Zainalabidin S. (2017). Roselle is cardioprotective in diet-induced obesity rat model with myocardial infarction. *Life Sciences*.

[43] Herrera-Arellano A., Miranda-Sánchez J., Ávila-Castro P. (2007). Clinical effects produced by a standardized herbal medicinal product of *Hibiscus sabdariffa* on patients with hypertension. A randomized, double-blind, lisinopril-controlled clinical trial. *Planta Medica*.

[44] Inuwa I., Ali B. H., al-Lawati I., Beegam S., Ziada A., Blunden G. (2012). Long-term ingestion of *Hibiscus sabdariffa* calyx extract enhances myocardial capillarization in the spontaneously hypertensive rat. *Experimental Biology and Medicine*.

[45] Buettner G. R. (2011). Superoxide dismutase in redox biology: the roles of superoxide and hydrogen peroxide. *Anti-Cancer Agents in Medicinal Chemistry*.

[46] Prakash P. A., Yogeswaran U., Chen S. M. (2009). A review on direct electrochemistry of catalase for electrochemical sensors. *Sensors*.

[47] Pérez-Torres I., Guarner-Lans V., Rubio-Ruiz M. E. (2017). Reductive stress in inflammation-associated diseases and the pro-oxidant effect of antioxidant agents. *International Journal of Molecular Sciences*.

[48] Yin G., Cao L., Xu P., Jeney G., Nakao M. (2011). Hepatoprotective and antioxidant effects *of Hibiscus sabdariffa* extract against carbon tetrachloride-induced hepatocyte damage in *Cyprinus carpio*. *In Vitro Cellular & Developmental Biology - Animal*.

[49] Mirochnitchenko O., Weisbrot-Lefkowitz M., Reuhl K., Chen L., Yang C., Inouye M. (1999). Acetaminophen toxicity: opposite effects of two forms of glutathione peroxidase. *Journal of Biological Chemistry*.

[50] Gauthaman K. K., Saleem M. T. S., Thanislas P. T. (2006). Cardioprotective effect of the *Hibiscus rosa sinensis* flowers in an oxidative stress model of myocardial ischemic reperfusion injury in rat. *BMC Complementary and Alternative Medicine*.

[51] Paglia D. E., Valentine W. N. (1967). Studies on the quantitative and qualitative characterization of erythrocyte glutathione peroxidase. *The Journal of Laboratory and Clinical Medicine*.

[52] Deponte M. (2013). Glutathione catalysis and the reaction mechanisms of glutathione-dependent enzymes. *Biochimica et Biophysica Acta (BBA) - General Subjects*.

[53] Lin C. Y., Huang C. S., Huang C. Y., Yin M. C. (2009). Anticoagulatory, antiinflammatory, and antioxidative effects of protocatechuic acid in diabetic mice. *Journal of Agricultural and Food Chemistry*.

[54] Lee C. H., Kuo C. Y., Wang C. J. (2012). A polyphenol extract of *Hibiscus sabdariffa* L. ameliorates acetaminophen-induced hepatic steatosis by attenuating the mitochondrial dysfunction *in vivo* and *in vitro*. *Bioscience, Biotechnology, and Biochemistry*.

[55] Vararattanavech A., Ketterman A. J. (2003). Multiple roles of glutathione binding-site residues of glutathione s-transferase. *Protein & Peptide Letters*.

[56] Soto M. E., Zuñiga-Muñoz A., Guarner Lans V., Duran-Hernández E. J., Pérez-Torres I. (2016). Infusion of *Hibiscus sabdariffa L*. modulates oxidative stress in patients with marfan syndrome. *Mediators of Inflammation*.

[57] Rybka J., Kupczyk D., Kędziora-Kornatowska K. (2011). Glutathione- related antioxidant defense system in elderly patients treated for hypertension. *Cardiovascular Toxicology*.

[58] Curtis J. M., Grimsrud P. A., Wright W. S. (2010). Downregulation of adipose glutathione S-transferase A4 leads to increased protein carbonylation, oxidative stress, and mitochondrial dysfunction. *Diabetes*.

[59] Banerjee D., Biggs M. L., Mercer L. (2013). Insulin resistance and risk of incident heart failure: cardiovascular health study. *Circulation: Heart Failure*.

[60] Sasaki R., Nishimura N., Hoshino H. (2007). Cyanidin 3-glucoside ameliorates hyperglycemia and insulin sensitivity due to downregulation of retinol binding protein 4 expression in diabetic mice. *Biochemical Pharmacology*.

[61] Hosseini A., Bakhtiari E., Mousavi S. H. (2017). Protective effect of *Hibiscus sabdariffa* on doxorubicin-induced cytotoxicity in H9c2 cardiomyoblast cells. *Iranian Journal of Pharmaceutical Research: IJPR*.

[62] Guéraud F., Atalay M., Bresgen N. (2010). Chemistry and biochemistry of lipid peroxidation products. *Free Radical Research*.

